# MicroRNA-148a inhibits breast cancer migration and invasion by directly targeting WNT-1

**DOI:** 10.3892/or.2022.8317

**Published:** 2022-04-13

**Authors:** Qian Jiang, Miao He, Meng-Tao Ma, Hui-Zhe Wu, Zhao-Jin Yu, Shu Guan, Long-Yang Jiang, Yan Wang, Da-Di Zheng, Feng Jin, Min-Jie Wei

Oncol Rep 35: 1425–1432, 2016; DOI: 10.3892/or.2015.4502

Subsequently to the publication of the above article, an interested reader drew to the authors attention that the data panel for the MDA-MB-231/migration/NC experiment in Fig. 2B on p. 1428 was strikingly similar to the data shown for the MDA-MB-231/invasion/Blank experiment in Fig. 2C, such that these data appeared to have been derived from the same original source. The authors have referred back to their original data, and realize that the data panel was selected incorrectly for Fig. 2B.

The corrected version of Fig. 2, showing the correct data for the MDA-MB-231/migration/NC experiment in Fig. 2B, is shown on the next page. The authors regret the error that was made during the preparation of this figure, and can confirm that the error in the assembly of this figure did not adversely affect the conclusions reported in the study. The authors are grateful to the Editor of *Oncology Reports* for allowing them the opportunity to publish a Corrigendum, and all the authors agree to this Corrigendum. Furthermore, they apologize to the readership for any inconvenience caused.

## Figures and Tables

**Figure 2.**
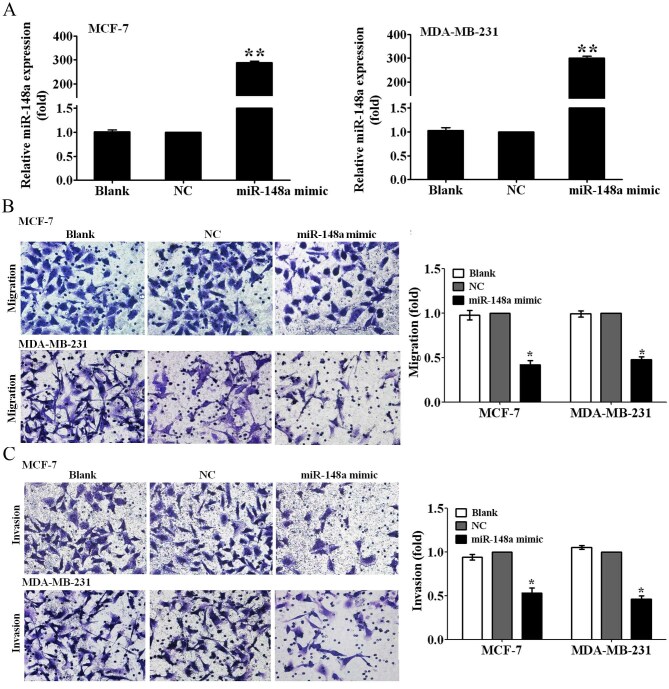
miR-148a suppresses the migration and invasion of breast cancer cells. (A) The relative expression of miR-148a was detected by qRT-PCR at 48 h after transfection with miR-148a mimic or NC in MCF-7 and MDA-MB-231 breast cancer cells. (B) The cell migration and (C) invasion abilities were measured by Transwell migration and invasion assays after transfection with miR-148a mimic or NC in MCF-7 and MDA-MB-231 cells. Cells migrating and invading the lower Transwell chambers were counted (magnification, ×200). The cell number migrating and invading the lower chambers in NC group was set as 1. Data are presented as mean ± SD from there independent experiments. *P<0.05, **P<0.01 vs. NC group.

